# Elimination of *Chlamydia muridarum* from the female reproductive tract is IL-12p40 dependent, but independent of Th1 and Th2 cells

**DOI:** 10.1371/journal.ppat.1011914

**Published:** 2024-01-02

**Authors:** Jordan A. Rixon, Kevin D. Fong, Claire Morris, Alana T. Nguyen, Claire E. Depew, Stephen J. McSorley

**Affiliations:** Department of Anatomy, Physiology and Cell Biology, School of Veterinary Medicine, University of California Davis, Davis, California, United States of America; Duke University School of Medicine, UNITED STATES

## Abstract

*Chlamydia* vaccine approaches aspire to induce Th1 cells for optimal protection, despite the fact that there is no direct evidence demonstrating Th1-mediated *Chlamydia* clearance from the female reproductive tract (FRT). We recently reported that T-bet-deficient mice can resolve primary *Chlamydia* infection normally, undermining the potentially protective role of Th1 cells in *Chlamydia* immunity. Here, we show that T-bet-deficient mice develop robust Th17 responses and that mice deficient in Th17 cells exhibit delayed bacterial clearance, demonstrating that *Chlamydia*-specific Th17 cells represent an underappreciated protective population. Additionally, Th2-deficient mice competently clear cervicovaginal infection. Furthermore, we show that sensing of IFN-γ by non-hematopoietic cells is essential for *Chlamydia* immunity, yet bacterial clearance in the FRT does not require IFN-γ secretion by CD4 T cells. Despite the fact that Th1 cells are not necessary for *Chlamydia* clearance, protective immunity to *Chlamydia* is still dependent on MHC class-II-restricted CD4 T cells and IL-12p40. Together, these data point to IL-12p40-dependent CD4 effector maturation as essential for *Chlamydia* immunity, and Th17 cells to a lesser extent, yet neither Th1 nor Th2 cell development is critical. Future *Chlamydia* vaccination efforts will be more effective if they focus on induction of this protective CD4 T cell population.

## Author summary

The mechanism that CD4 T cells use to mediate clearance of *Chlamydia* infection in the female reproductive tract remains unclear. Contrary to prevailing ideas, previous work from our lab demonstrated that the Th1 subset characterized by T-bet expression is not required for clearance. Here, we explore other T helper subsets and determine that Th2 cells are likewise not required while Th17 cells contribute to the early phase of infection clearance. Ultimately, the cytokine subunit IL-12p40 plays a substantial role in driving an effective CD4 T cell response that is not neatly defined by canonical CD4 subset Th1, Th2, or Th17 identity. Vaccine development thus should focus on this IL-12p40-driven CD4 T cell response rather than relying on markers of classical T helper subsets.

## Introduction

*Chlamydia trachomatis* is a common cause of genital tract infection and remains an important public health problem in the US [1]. *Chlamydia* is now the most common notifiable bacterial infection reported to the Centers for Disease Control and Prevention (CDC), with over 1.6 million cases in 2021 [1]. The bulk of these genital tract infections are initially asymptomatic in young females (15–24 years old) but can cause significant reproductive harm if not detected and treated [2]. Chronic *Chlamydia* infections are a major cause of pelvic inflammatory disease, ectopic pregnancy, and other serious reproductive complications among women of child bearing age [2]. Unfortunately, current tools for preventing *Chlamydia trachomatis* infection do not include an effective vaccine [3]. Early attempts to develop a whole cell or live vaccine for *Chlamydia* trachoma encountered difficulty when clinical trials detected increased pathological outcomes for a subset of vaccinated individuals [4]. Although the validity of these adverse vaccine events has been questioned [5], the perception is that whole cell *Chlamydia* vaccines could cause reproductive harm to healthy young adults. Understanding the pathogenesis of Chlamydial disease and protective immune responses in animal models should uncover new prophylactic approaches.

Mouse models have provided an excellent opportunity to interrogate immunological responses to *Chlamydia* infection of the female reproductive tract (FRT) [6–8]. Indeed, vaccine-mediated protection and certain elements of immune suppression have been detected within the murine reproductive tract [9], providing confidence that this animal model accurately reproduces clinical observations in humans. Cervicovaginal infection of mice with *Chlamydia muridarum* initiates an ascending reproductive tract infection of epithelial cells [10], causing severe pathology similar to human *Chlamydia trachomatis* infection [2,11]. Over the past few decades, this mouse model has been used to uncover aspects of basic immunity to *Chlamydia* genital infection.

CD4 T cells are a critical component of the host immune response to *Chlamydia* within the FRT, although B cells can also contribute to protection [12,13]. SCID and RAG-deficient mice suffer lethal disseminated infection, while TCRα-deficient and MHC class II-deficient mice each fail to resolve primary *Chlamydia* infection of the FRT [14–18]. In marked contrast, mice lacking B cells or CD8 T cells resolve a primary *Chlamydia* infection, though B cell-deficient mice develop a transient systemic infection not detected in wild-type mice [19]. While CD4 T cell helper activity appears to be the foundation of *Chlamydia* immunity, it has been extremely difficult to identify the effector module responsible for bacterial killing *in vivo*. Activated CD4 T cells are generally thought to develop a limited range of effector responses summarized by basic effector modules (Th1/Th2/Th17, Tfh, Treg). Each of these modules expresses unique effector molecules that allow for coordination of innate cells to combat different classes of pathogen [20]. To mediate immunity to pathogenic bacteria, the critical effector modules include Th1 cells to combat bacteria growing inside macrophage vacuoles and Th17 cells to eradicate extracellular bacteria. Th1 cells are usually defined by the expression of the master transcription factor, T-bet, plus secretion of IFN-γ, a key cytokine that activates macrophages for increased intracellular killing [21,22]. In contrast, Th17 cells are defined by RORγt expression and secrete IL-17, a cytokine that initiates neutrophil recruitment for phagocytosis of extracellular bacteria [23–25]. While Th1 and Th17 cells combat bacterial infections, Th2 cells express GATA3 and secrete cytokines that can coordinate mast cell and eosinophil defense against helminths [26]. Although this simple T helper framework allows a basic understanding of host responses to many pathogens, the exact module responsible for *Chlamydia* killing in the FRT has been difficult to identify.

CD4 T cells in human *Chlamydia* infection secrete significant levels of IFN-γ [27,28]. Similarly, CD4 T cells recovered from the FRT of *Chlamydia*-infected mice secrete IFN-γ [19], while mice genetically deficient in IFN-γ or IFN-γR suffer from an overwhelming systemic infection [29,30]. Thus, both CD4 T cells and IFN-γ production are essential components of *Chlamydia* immunity, making it tempting to attribute *Chlamydia* host defense to the activity of Th1 cells [11,31–33]. However, the notion that CD4 Th1 cell production of IFN-γ mediates Chlamydial defense is not substantiated by experimental data. First, it has been noted by several groups that IFN-γ is secreted by a wide variety of innate lymphocytes within the *Chlamydia*-infected FRT [34,35]. Thus, the extreme susceptibility of mice lacking IFN-γ or IFN-γR might be due to non-CD4 T cell production of IFN-γ. Furthermore, recent work from our laboratory demonstrated that *Chlamydia*-specific CD4 T cells express low levels of the Th1 transcription factor T-bet and mice lacking T-bet expression resolve infection normally [30]. Together, these data suggest that while IFN-γ is a necessary component of *Chlamydia* immunity, FRT protection is not mediated by classical Th1 cells.

In this current study, we reexamine the role of Th2 and Th17 cells in *Chlamydia* immunity and investigate whether redundancy exists in the contribution of Th1 and Th17 cells within the FRT. Our data eliminate a requirement for Th2 cells, but uncover an essential role for Th17 cells in accelerated bacterial clearance. Additionally, we show that IL-12p40 and sensing of IFN-γ by non-hematopoietic cells are essential to *Chlamydia* clearance, while CD4 T cell production of IFN-γ is not required. Compensation between Th1 and Th17 responses was insufficient to explain the protective role of CD4 T cells in *Chlamydia* clearance. Together, our data suggest that an IL-12p40-dependent protective CD4 module emerges to combat *Chlamydia* infection that is distinct from well-described Th1 and Th17 effectors.

## Results

### Mice deficient in Th2 cells clear *Chlamydia* infection similarly to wild-type mice

**Fig 1:**
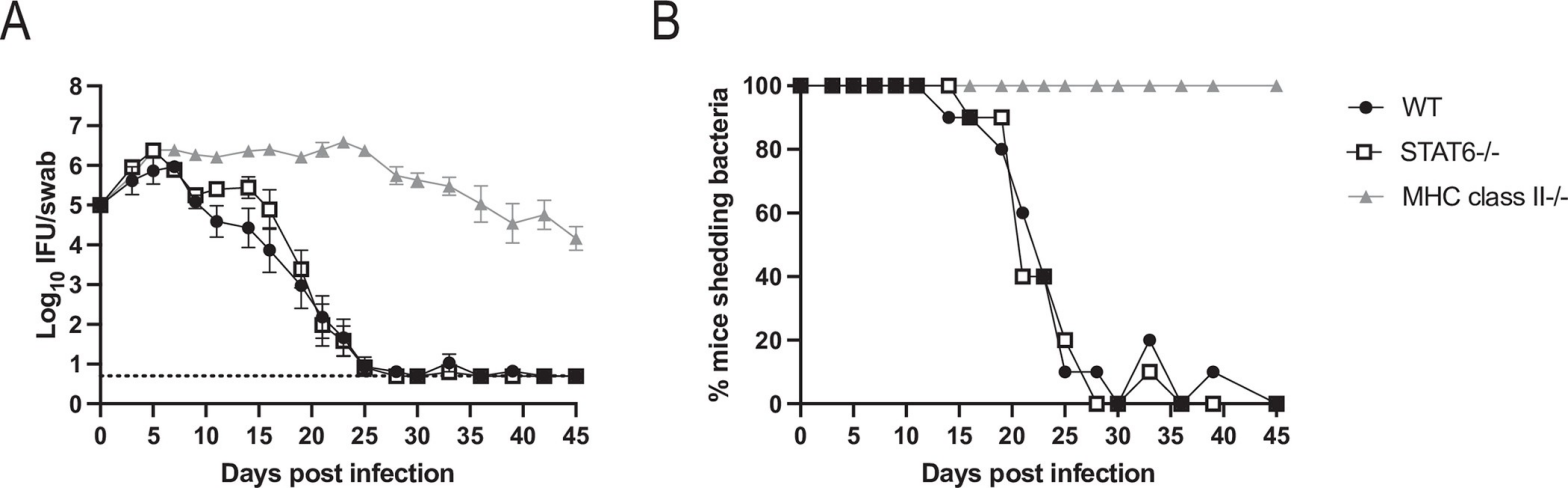
STAT6-deficient mice do not show any deficiency in *Chlamydia* clearance. (A) IFUs isolated from vaginal swabs at various time points after infection. For wild-type mice n = 10, STAT6-deficient mice n = 10, and MHC class II-deficient mice n = 5. Data is combined from two experiments, with the MHC class II-deficient group included in one experiment. Graph is displayed as mean ± SEM. Wild-type versus STAT6-deficient groups are not significantly different. For wild-type versus MHC class II-deficient groups p<0.05 on days 9–45. For STAT6-deficient versus MHC class II-deficient groups p<0.05 on days 7–45 (mixed-effects analysis). Dashed line represents limit of detection. (B) Data from A expressed as the percent of mice with culture positive vaginal swabs.

A consensus has emerged that CD4-mediated immunity to *Chlamydia* in the FRT is mediated by Th1 cells [11,31–33], which are usually defined by the expression of T-bet and IFN-γ [36]. However, we recently reported that mice with CD4 T cells lacking T-bet, or mice completely deficient in T-bet, can each resolve *Chlamydia* FRT infection similar to wild-type controls [30], suggesting the involvement of other CD4 effector modules. A previous study reported Th2-like responses in patients infected with *Chlamydia* [37], raising the possibility that type 2 responses could participate in bacterial clearance. Indeed, in the mouse model, Th2 responses have been correlated with protection against pathology, while IL-13 was associated with both clearance and susceptibility to infection [38–40]. Although the transcription factor GATA3 regulates Th2 development, *Gata3* null mutant mice are embryonic lethal, thus we examined *Chlamydia* infection of STAT6-deficient mice which lack Th2 development [41,42]. *Chlamydia*-infected STAT6-deficient mice shed bacteria from the FRT with similar kinetics to wild-type mice (Fig 1A). Furthermore, the kinetics of bacterial clearance from the FRT was similar in STAT6-deficient and wild-type mice whether this was expressed as IFU isolated from vaginal swabs or the percentage of culture positive mice at any given time (Fig 1A and 1B). Together, these data demonstrate that Th2 development is not a critical component of *Chlamydia* clearance from the FRT of infected C57BL/6 mice.

### *Chlamydia*-specific CD4 T cells shift to a Th17 signature in T-bet-deficient mice

**Fig 2:**
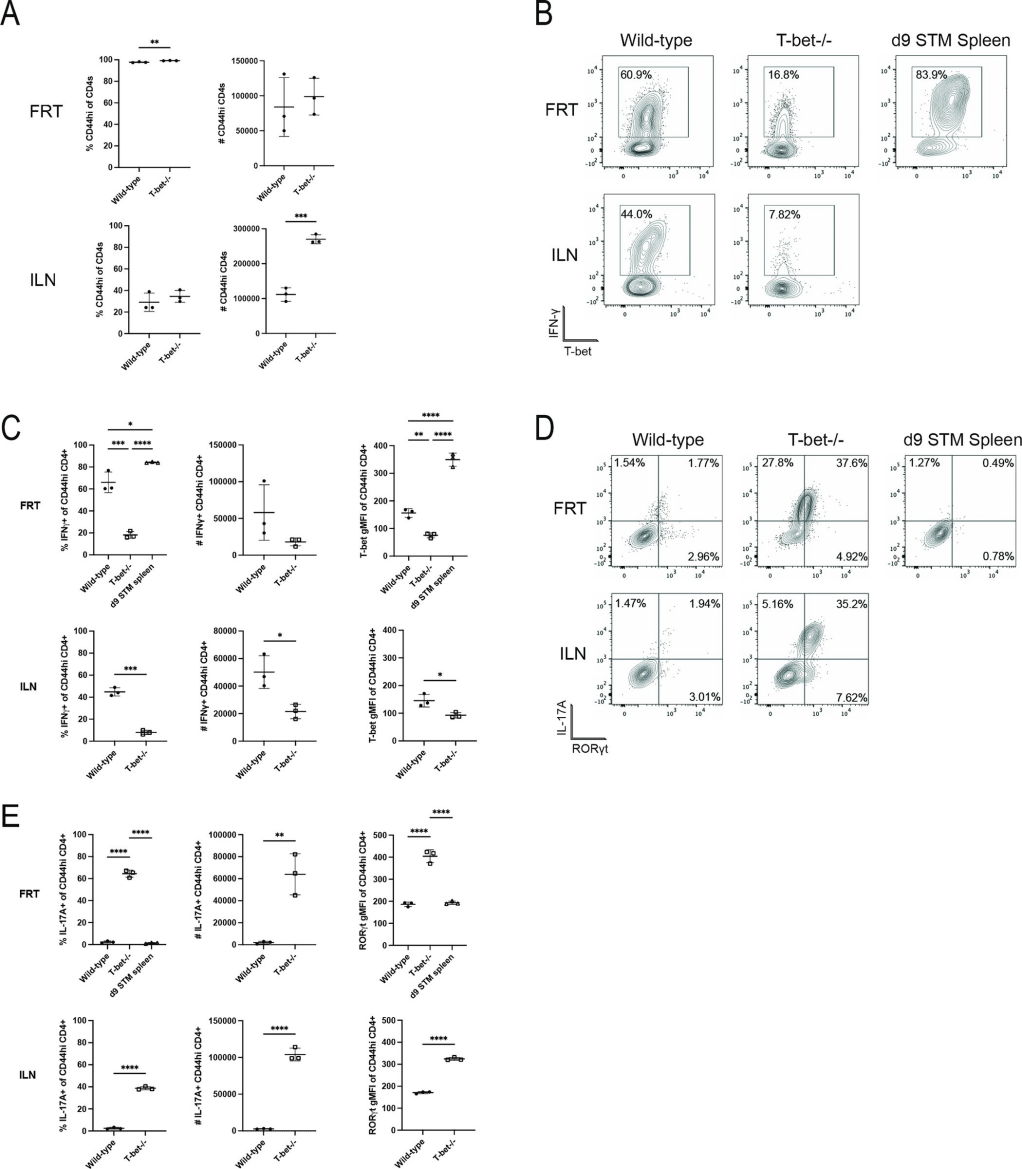
T-bet deficient mice display a significant shift towards Th17 responses. Lymphocytes isolated from the FRT and ILN were stimulated with PMA and ionomycin with Brefeldin A before staining for flow cytometry. Results are gated on CD4+ CD44hi cells. (A) Percentage and number of total activated CD4 T cells isolated from FRT and ILN. (B) Expression of Th1 markers T-bet and IFN-γ. (C) Summary graphs from B. (D) Flow cytometry plots showing the expression of Th17 markers RORγt and IL-17A. (E) Summary graphs from D. All graphs are displayed as mean ± SD. Data is representative of two experiments.

The majority of CD4 T cells in the FRT of *Chlamydia*-infected mice secrete high levels of IFN-γ but express unusually low levels of T-bet compared to a classical Th1 responses in *Salmonella-*infected mice (Fig 2B and 2C, and flow gating shown in S1). Indeed, T-bet expression in CD4 T cells is low in the FRT and draining iliac lymph nodes (ILN), yet IFN-γ secretion is particularly high in the FRT (Fig 2B and 2C). Th17 cells (RORγt and IL-17A) represent a very small fraction of the CD4 T cell population in *Chlamydia*-infected C57BL/6 mice (Fig 2D and 2E). However, CD4 T cells isolated from the FRT of T-bet-deficient mice displayed marked differences in cytokine production compared to wild-type mice. Specifically, in T-bet-deficient mice the proportion of IFN-γ producing CD4 T cells fell to less than a third of that detected in wild-type mice (Fig 2B and 2C), while RORγt and IL-17A expression increased from less than 5% to around 60% in the FRT and 40% in the ILN (Fig 2D and 2E). Thus, despite the fact that there is no detectable difference in bacterial shedding between wild-type and T-bet-deficient mice [30], a prominent increase in Th17 type development is observed in the absence of T-bet. It should be noted that this expansion in Th17 cell response was not associated with enhanced pathology in T-bet-deficient mice [30].

### Mice lacking IL-12p40 have a marked deficiency in *Chlamydia* clearance

**Fig 3:**
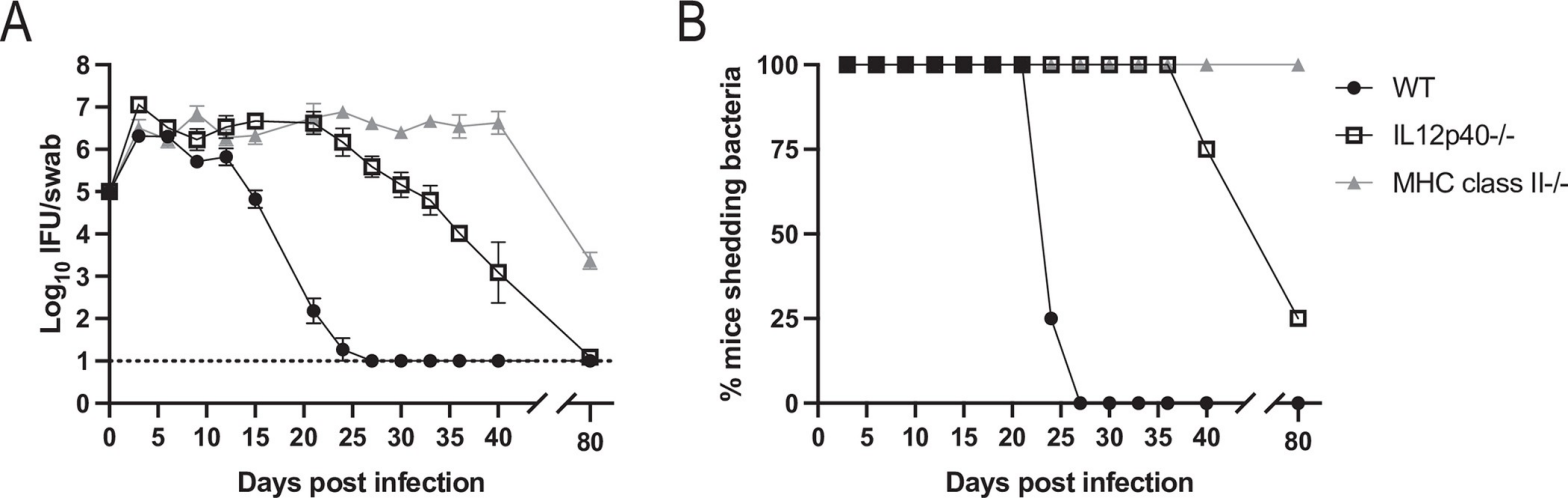
IL-12p40-deficient mice exhibit a severe delay in *Chlamydia* clearance in the FRT. (A) IFUs isolated from vaginal swabs at various time points after infection. n = 4 for all groups. Graph is displayed as mean ± SEM. For wild-type versus IL-12p40-/- groups, p<0.05 on days 3 and 15–36. For wild-type versus MHC class II-deficient groups, p<0.05 on days 9 and 15–80. For IL12p40-/- versus MHC class II-deficient groups, p<0.05 on days 27–80 (mixed-effects model). (B) Data from A expressed as the percent of mice with culture positive vaginal swabs. Data is representative of two experiments.

Given increased development of Th17 cells in the absence of T-bet, we decided to examine the role of IL-12p40 in *Chlamydia* clearance. IL-12p40 is a component of the Th1 and Th17-related cytokines IL-12 and IL-23, meaning that IL-12p40-deficient mice have Th1 and Th17 deficiencies [43]. Indeed, mice lacking IL-12p40 exhibited a severe defect in *Chlamydia* clearance with a marked delay in the ability to resolve FRT infection (Fig 3A and 3B). We also observed a reduced capacity for IFN-γ from the CD4 T cells in the FRT and ILN of these mice to approximately half of that of wild-type mice (S2 Fig). The delay in FRT clearance by IL-12p40-deficient mice was somewhat unexpected since a prior study showed that IL-12p40-deficient mice have no deficiency resolving *Chlamydia* infection [44]. This discrepancy was possibly explained by the difference in relative pathogenicity of *Chlamydia* strains used by these studies. Indeed, when mice were infected with a less pathogenic Nigg strain, we replicated previously published data showing no requirement for IL-12p40, yet a clear requirement for IL-12p40 was still observed using the more pathogenic strain derived from ATCC stock used in our study (S3A and S3B).

### Mice lacking Th17 responses display an impaired ability to resolve *Chlamydia* infection

**Fig 4:**
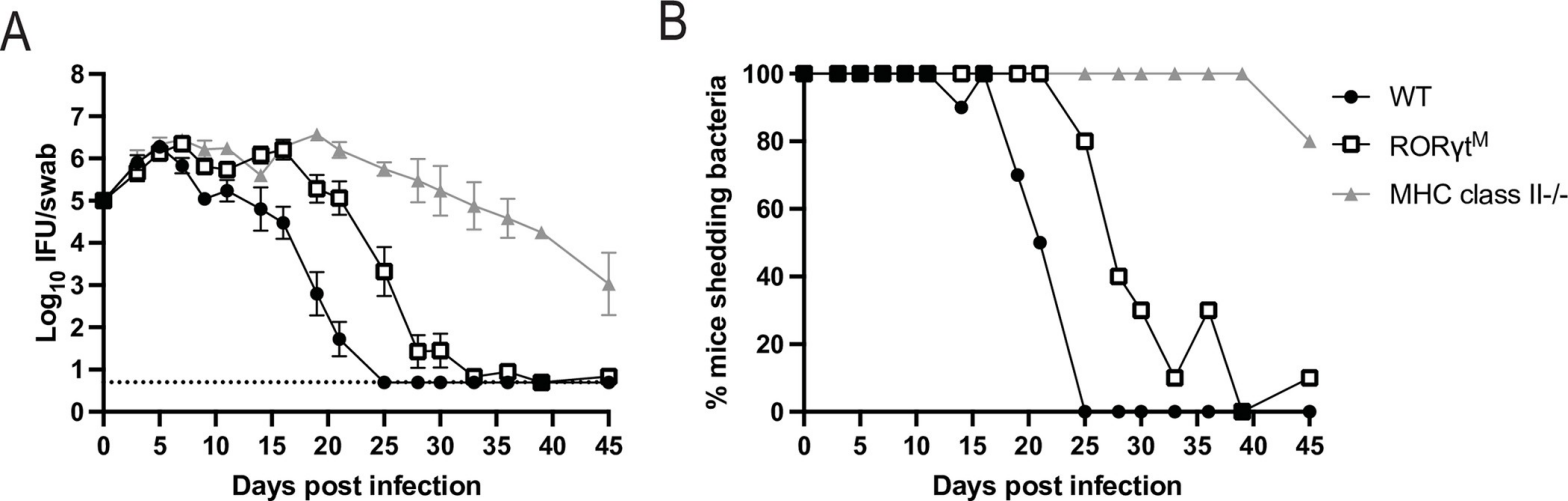
RORγt mutant mice have a delay in *Chlamydia* clearance. (A) IFUs isolated from vaginal swabs at various time points after infection. For wild-type mice n = 10, RORγt mutant (RORγt^M^) mice n = 10, and MHC class II-/- mice n = 5. Data is combined from two experiments, MHC class II-/- group was included in one experiment. Graph is displayed as mean ± SEM. For wild-type versus RORγt^M^ groups, p<0.05 on days 9 and 16–25. For wild-type versus MHC class II-deficient groups, p<0.05 on days 7–11 and 16–39. For RORγt^M^ versus MHC class II-deficient groups, p<0.05 on days 11, 19, and 25–39 (2-way ANOVA). (B) Data from A expressed as the percent of mice with culture positive vaginal swabs.

Since Th17 cells were the major CD4 T cell response in T-bet-deficient mice, we examined whether this effector subset was involved in bacteria clearance. RORγt null mice have impaired lymph node and T cell development, reducing their utility for examining peripheral Th17 deficiency [45,46]. However, RORγt mutant mice (RORγt^M^) have a two amino acid mutation that allows lymph node and T cell development, but impedes development of Th17 responses [47]. When these mice were infected with *Chlamydia* they exhibited a significant delay in bacterial clearance compared to wild-type mice (Fig 4A and 4B). However, RORγt mutant mice eventually cleared *Chlamydia* from the FRT, demonstrating that non-Th17 effector mechanisms also participate in the resolution of *Chlamydia* infection. In conclusion, these data show that the Th17 responses that emerge in the absence of T-bet are required for efficient clearance of *Chlamydia* from the FRT.

**Fig 5:**
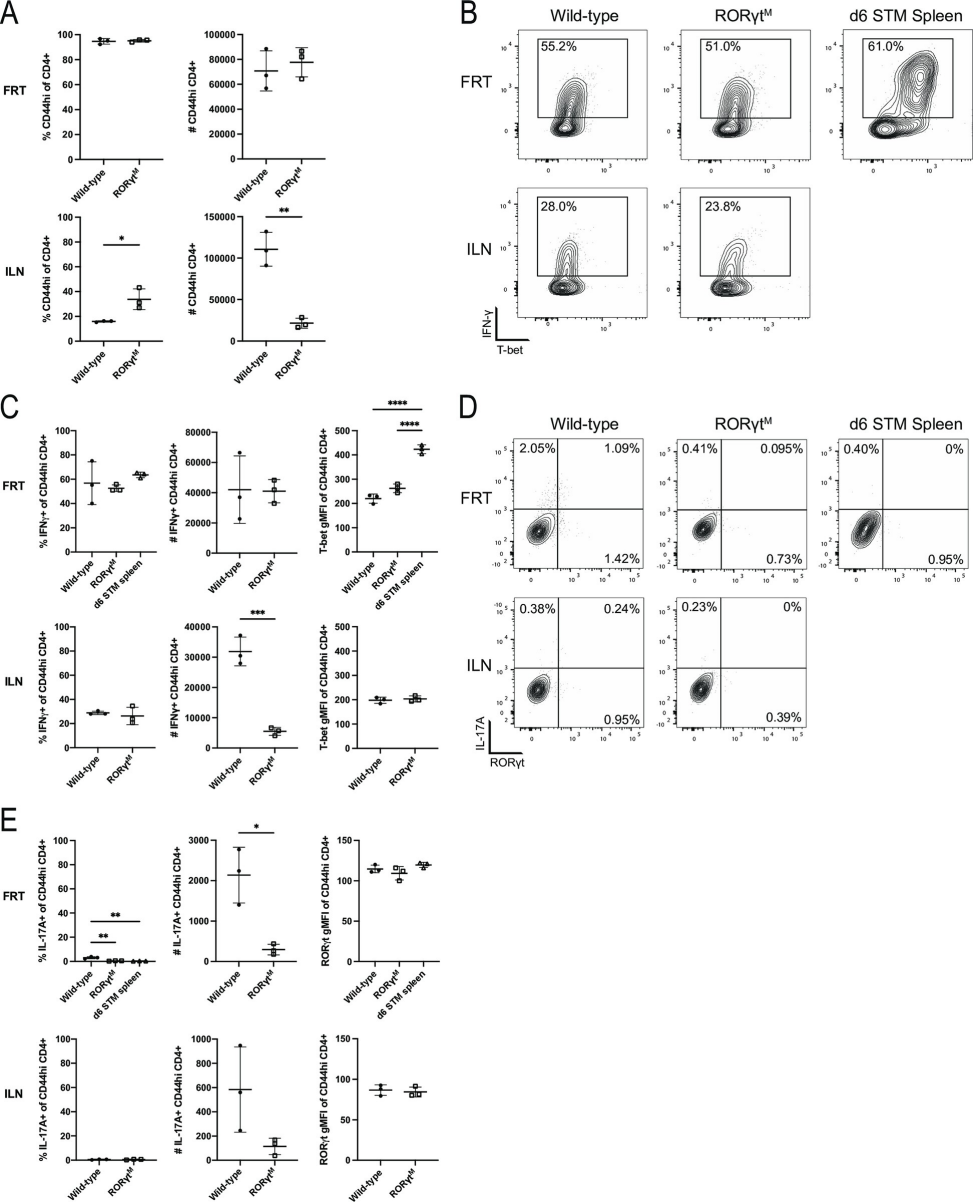
CD4 T cells from RORγt mutant mice have a similar expression pattern of Th1 and Th17 markers as wild-type mice. Lymphocytes isolated from the FRT and ILN were stimulated with PMA and ionomycin with Brefeldin A before staining for flow cytometry. Results are gated on CD4+ CD44hi cells. (A) Percentage and number of total activated CD4 T cells isolated from FRT and ILN. (B) Expression of Th1 markers T-bet and IFN-γ. (C) Summary graphs from B. (D) Flow cytometry of expression of Th17 markers RORγt and IL-17A. (E) Summary graphs from D. All graphs are displayed as mean ± SD. Data is representative of two experiments.

In order to examine this effector mechanism in more detail, we compared CD4 T cells responding to *Chlamydia* in the FRT of RORγt mutant mice and wild-type mice. There was a small reduction in the number of activated CD4 T cells in the ILN of RORγt mutant mice, but this did not influence the total number of CD4 T cells in the FRT (Fig 5A). The proportion of CD4 T cells expressing IFN-γ was similar in the FRT and ILN of RORγt mutant and wild-type mice (Fig 5B and 5C). As expected, the low level of IL-17A-producing CD4 T cells detected in the FRT of wild-type mice was further diminished in RORγt mutant mice (Fig 5D and 5E). Overall, these data show that RORγt mutant mice display modest differences in CD4 development compared to wild-type mice. Given these minor differences in cytokine production between CD4 effector responses in wild-type and RORγt mutant mice, we used transcriptional analysis to get a deeper understanding. We used bulk RNA-seq to interrogate overall gene signatures in CD4 T cells isolated from the FRT of wild-type and RORγt mutant mice, 17 days post infection. At this time point, wild-type mice were actively clearing *Chlamydia* infection while RORγt mutant mice retained a high rate of bacterial shedding (Fig 4). The top ten differentially expressed genes between wild-type and RORγt mutant CD4 T cells are displayed in Table 1. Only three genes, *Il1r1*, *Il17re*, and *Ramp1*, had adjusted p-values below 0.05, further indicating that these CD4 responses remain similar. With *Il22* being the fourth ranked gene (p-value of 0.087), along with *Il1r1* and *Il17re*, our transcriptional data support our flow cytometry findings showing that RORγt mutant CD4 T cells have lower Th17 responses. Overall, these data show that Th17 cells are critical for efficient bacterial clearance in the FRT, despite the fact that they represent a small fraction of the overall CD4 T cell response to *Chlamydia*.

**Table 1:** Bulk RNA-seq on CD4 T cells from the FRTs of RORɣt mutant mice show few differences from wild-type mice.

Gene Name	Log2 Fold Change	Average Expression	Adjusted p value	Gene Description
*Il1r1*	-3.08	5.26	**0.0071**	interleukin 1 receptor, type I [Source:MGI Symbol;Acc:MGI:96545]
*Il17re*	-2.53	5.43	**0.0148**	interleukin 17 receptor E [Source:MGI Symbol;Acc:MGI:1889371]
*Ramp1*	-3.13	4.56	**0.0148**	receptor (calcitonin) activity modifying protein 1 [Source:MGI Symbol;Acc:MGI:1858418]
*Il22*	-5.48	2.58	0.0876	interleukin 22 [Source:MGI Symbol;Acc:MGI:1355307]
*Ighg2c*	2.73	2.29	0.1707	immunoglobulin heavy constant gamma 2C [Source:MGI Symbol;Acc:MGI:2686979]
*Myl12b*	-0.97	6.71	0.2665	myosin, light chain 12B, regulatory [Source:MGI Symbol;Acc:MGI:107494]
*Nrgn*	-3.04	4.03	0.3077	neurogranin [Source:MGI Symbol;Acc:MGI:1927184]
*Tmem176a*	-2.13	5.33	0.3077	transmembrane protein 176A [Source:MGI Symbol;Acc:MGI:1913308]
*Ccl5*	0.85	7.45	0.3077	chemokine (C-C motif) ligand 5 [Source:MGI Symbol;Acc:MGI:98262]
*Ccdc66*	1.87	4.15	0.308	coiled-coil domain containing 66 [Source:MGI Symbol;Acc:MGI:2443639]

At 17 days post infection, CD4+ T cells were FACS sorted from infected FRTs of RORɣt mutant and wild-type mice and underwent RNA sequencing. This table lists differential gene expression analysis of RORɣt mutants compared to wild-type, including the top 10 genes as ordered by adjusted p-value. Bold values are p<0.05. Average expression is across all samples in log2 counts per million reads and the adjusted p-value uses the Benjamini-Hochberg false discovery rate.

### Blocking Th1 and Th17 pathways concurrently does not prevent bacterial clearance

**Fig 6:**
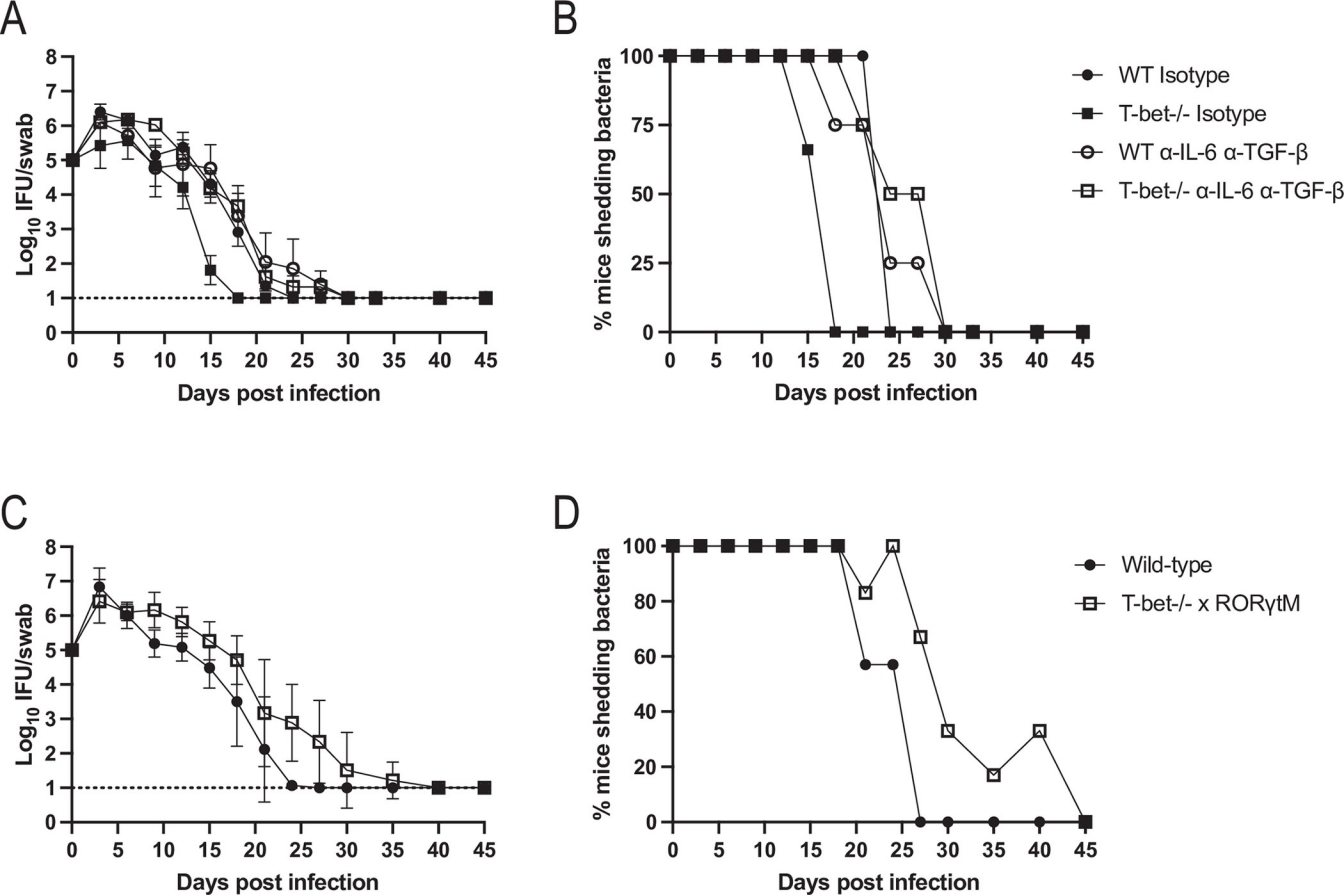
Depleting Th1 and Th17 cells does not impact FRT clearance of *Chlamydia*. (A and C) IFUs isolated from vaginal swabs at various time points after infection. Graphs are displayed as mean ± SEM. (B and D) Data from A and C expressed as the percent of mice with culture positive vaginal swabs. (A and B) Wild-type or T-bet-/- mice were given isotype or depleting antibodies for IL-6 and TGF-β. (C and D) T-bet-/- mice were bred with RORγt mutant mice and infected with *Chlamydia*. (A) n = 3 for isotype treated groups, n = 4 for antibody treated groups. All time points and comparisons are not significantly different except day 18 for wild-type isotype versus T-bet-/- isotype and day 18 for T-bet-/- isotype versus T-bet-/- anti-IL-6 anti-TGF-β (2-way ANOVA). (B) Data is combined from two experiments. n = 7 for wild-type, n = 6 for T-bet-/- x RORγt^M^. p<0.05 on days 9–12 and 24 (mixed-effects analysis).

Although Th17 cells are required for rapid resolution of *Chlamydia* infection (Fig 4), our data also suggests that complete clearance of *Chlamydia* occurs in mice lacking either of the three common CD4 modules (Th1, Th2 and Th17). Given the high level of IFN-γ production by CD4 T cells in wild-type mice, the compensatory IL-17 response in T-bet-deficient mice, and the severe delay in clearance noted in IL-12p40-deficient mice, we hypothesized that Th1-like and Th17-like cells provide overlapping protection with either response being sufficient for bacterial clearance. In order to examine this possibility, we use two approaches to limit Th1 and Th17 programs in infected mice. First, we used neutralizing antibodies against IL-6 and TGF-β to inhibit Th17 development in T-bet-deficient mice while control mice were administered isotype antibodies. Neither wild-type or T-bet-deficient mice administered IL-6/TGF-β depleting antibodies displayed evidence of delayed bacterial clearance from the FRT (Fig 6A and 6B). Thus, a reduction in Th1/Th17 responses was insufficient to block *Chlamydia* clearance from the FRT. As a complementary approach, we backcrossed RORγt mutant mice to a T-bet-deficient background to generate mice with a combined Th1 and Th17 deficiency. Flow cytometry analysis confirmed a drastic reduction in IL-17A production in these mice compared to T-bet-deficient mice, though it was not completely eliminated (S2 Fig). Again, bacterial clearance in these double-deficient mice did not deviate dramatically from C57BL/6 mice, where bacterial burdens began to reduce in the second week of infection (Fig 6C). However, there was a delay in clearing bacteria later during the third week of infection, on day 24 (Fig 6D), consistent with our finding that Th17 cells are required for rapid resolution. Together, these data fail to support a model where Th1 and Th17 responses can interchangeably compensate for each other during clearance of *Chlamydia*. It is important to note that this outcome is surprising given the clear requirement for IL-12p40 in bacterial clearance (Fig 3).

### Non-hematopoietic cell sensing of IFN-γ is critical for *Chlamydia* clearance

**Fig 7:**
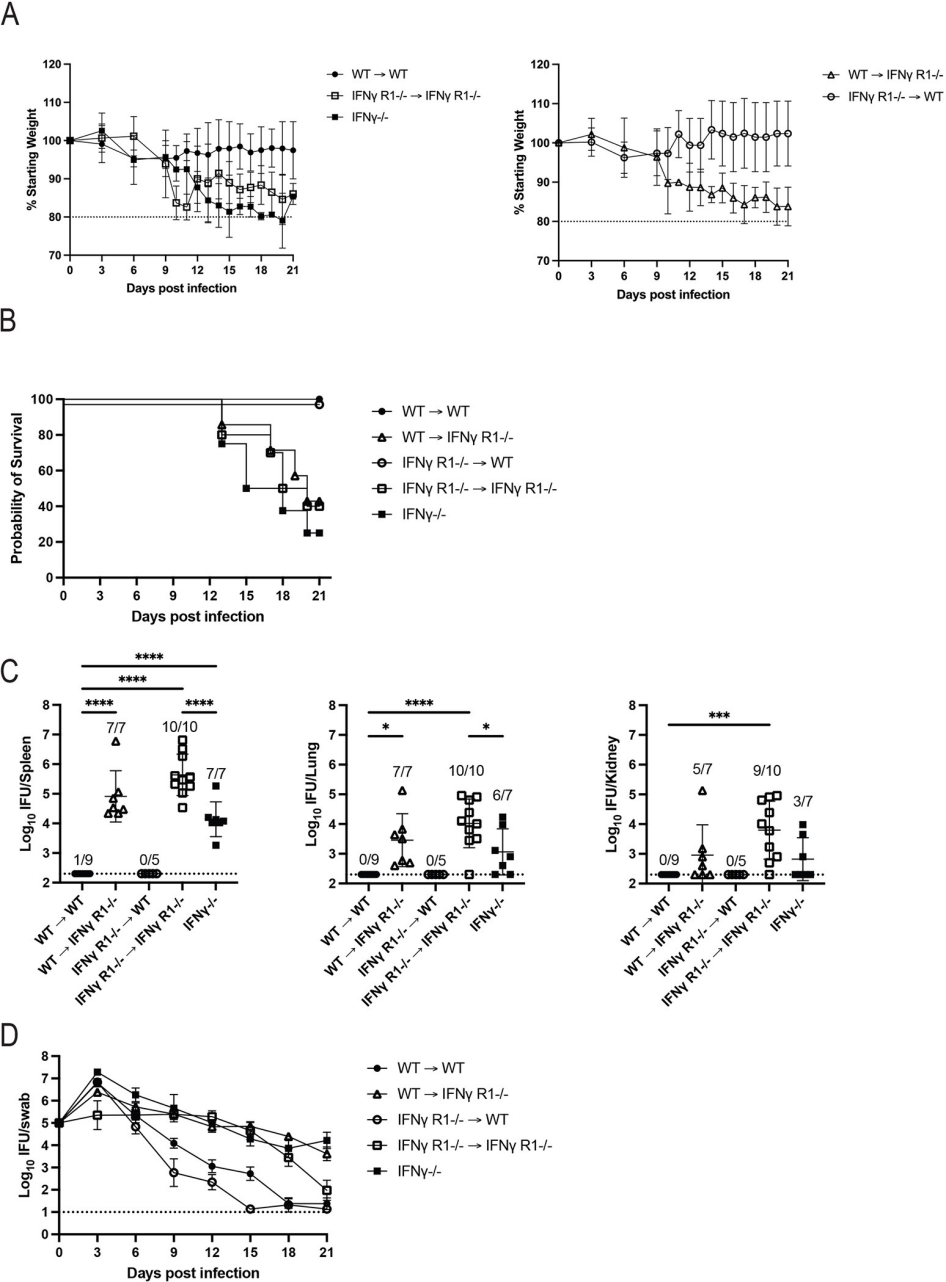
Bone marrow chimera mice require expression of IFN-γ receptor by the recipient host tissues, but not donor bone marrow cells to control systemic *Chlamydia* infection. Four groups of bone marrow chimera mice were generated as follows: CD45.1 wild-type bone marrow was transferred into CD45.2 wild-type recipients (WT→WT), CD45.1 wild-type bone marrow was transferred into CD45.2 IFNγR1-/- recipients (WT→IFNγ R1-/-), CD45.2 IFNγR1-/- bone marrow was transferred into CD45.1 wild-type recipients (IFNγ R1-/-→WT), and CD45.2 IFNγR1-/- bone marrow was transferred into CD45.2 IFNγR1-/- recipients (IFNγ R1-/-→IFNγ R1-/-). These groups were synchronized and infected alongside an additional, unmanipulated group of IFNγ-/- mice. The weight of each mouse was monitored over the course of the experiment and mice were euthanized before day 21 post infection if their weight was below 80% of the starting value or their condition otherwise became too severe. All remaining mice were euthanized at day 21. Upon euthanasia, spleen, lung, and kidneys were harvested for counting *Chlamydia* burdens. (A) Graphs show the percentage of starting weight of each mouse over time ± SD. Controls and experimental groups are shown in separate plots for readability. WT→WT versus IFNγ R1-/-→WT is not significantly different. For WT→WT versus WT→IFNγ R1-/-, p<0.05 on days 11 and 14–20. For WT→WT versus IFNγ R1-/-→IFNγ R1-/-, p<0.05 on days 6, 10–11, and 16–21. For WT→WT versus IFNγ-/-, p<0.05 on days 12–19 and 21. For WT→IFNγ R1-/- versus IFNγ R1-/-→WT, p<0.05 on days 11, 14, 17, and 20–21 (mixed-effects analysis). (B) Survival curve. With Bonferroni correction for multiple comparisons, WT→WT versus IFNγ R1-/-→WT is not significantly different while all other groups compared to WT→WT are significant. (C) Bacterial load measured in each organ at the time of euthanasia ± SD (1-way ANOVA). (D) IFUs isolated from vaginal swabs over the course of infection ± SEM. For WT→WT versus IFNγ R1-/-→WT, p<0.05 on day 15. For WT→WT versus WT→IFNγ R1-/-, p<0.05 on days 9–21. For WT→WT versus IFNγ R1-/-→IFNγ R1-/-, p<0.05 on days 9–18. For WT→WT versus IFNγ-/-, p<0.05 on days 12–21 (mixed-effects analysis). Data is combined from two experiments, total n are 9 for WT→WT, 7 for WT→IFNγ R1-/-, 5 for IFNγ R1-/-→WT, 10 for IFNγ R1-/-→IFNγ R1-/-, and 8 for IFNγ-/-.

Studies by our laboratory and others show that IFN-γ- and IFN-γ-R-deficient mice display a major deficiency in resolving *Chlamydia* infection and can develop disseminated infection that often proves to be fatal [29,30,34]. Given the systemic nature of these infections, we hypothesized that *Chlamydia* grows in phagocytes that require IFN-γ signaling to control bacterial growth, similar to models of *Salmonella* immunity [48]. An alternative hypothesis would be that FRT epithelial cells, rather than phagocytes, need IFN-γ signaling to limit local FRT infection, reducing the potential for systemic spread of bacteria. These models are not mutually exclusive, but present two different mechanisms that explain IFN-γ-mediated control of disseminated infections. To examine this issue, we generated bone marrow chimeric mice where hematopoietic cells or non-hematopoietic cells lack the IFN-γR and infected these mice with *Chlamydia*. As expected, wild-type mice reconstituted with wild-type bone marrow retained a normal body weight (Fig 7A, left), displayed no overt signs of systemic disease (Fig 7B), had low levels of systemic bacteria (Fig 7C), and resolved FRT *Chlamydia* infection (Fig 7D). In contrast, IFN-γR-deficient mice reconstituted with IFN-γR-deficient bone marrow lost a significant amount of body weight (Fig 7A, left), eventually succumbed to infection with similar kinetics to IFN-γ-deficient controls (Fig 7B), had bacteria in the spleen, lung, and kidneys (Fig 7C), and had delayed resolution of FRT infection (Fig 7D). Interestingly, wild-type mice reconstituted with IFN-γ receptor-deficient bone marrow, did not lose body weight (Fig 7A, right), exhibit signs of sickness or display evidence of systemic infection (Fig 7B and 7C), and controlled bacterial growth within the FRT (Fig 7D). Thus, resolution of *Chlamydia* infection does not require sensing of IFN-γ by bone marrow-derived cells. In marked contrast, IFN-γ receptor-deficient mice reconstituted with wild-type bone marrow suffered weight loss, early mortality, and systemic dissemination (Fig 7A right, 7B and 7C), clearly demonstrating that IFN-γ signaling by non-hematopoietic cells is essential for *Chlamydia* clearance.

### CD4 T cell expression of IFN-γ is not required for *Chlamydia* clearance

**Fig 8:**
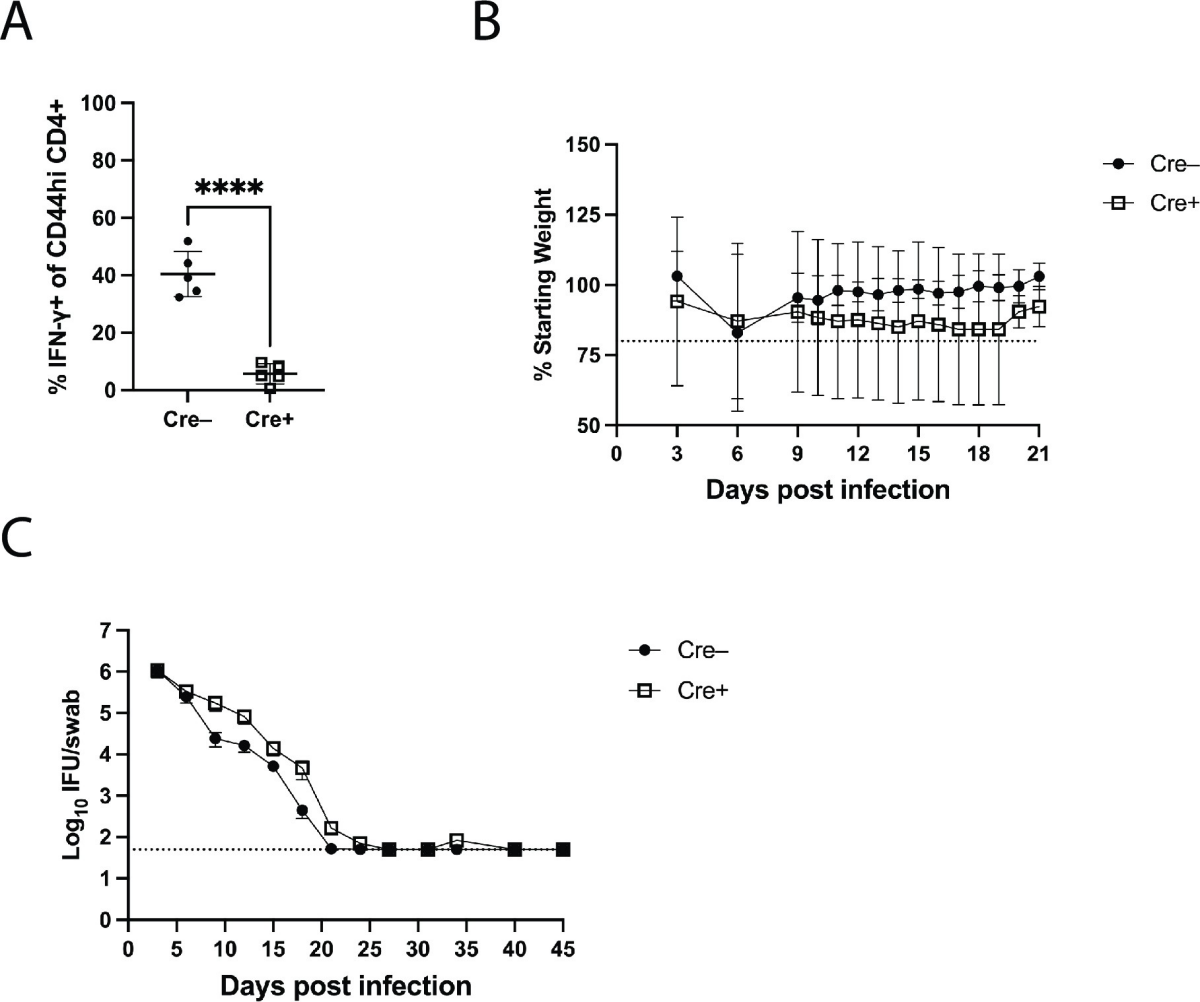
CD4 T cell derived IFN-γ is not required for bacterial clearance in the FRT. Two groups of bone marrow chimeric mice were generated: CD4-Cre+ IFN-γ floxed bone marrow transferred into CD45.1 recipients (Cre+, n = 11) and IFN-γ floxed bone marrow transferred into CD45.1 recipients (Cre-, n = 10). Mice were synchronized and weighed before infection. Five mice from each group were euthanized at day 21 and spleen, lung, and kidneys were harvested for counting *Chlamydia* burdens, and the FRT was harvested to assess IFN-γ expression in CD4 T cells. Vaginal swabs were taken periodically from all mice up to day 21 and in remaining mice after day 21. Four Cre+ mice died between day 21 and end of experiment (days 24, 27, 31, and 40). (A) Expression of IFN-γ in CD4+ T cells from the FRT. Results are gated on CD4+ CD44hi cells. (B) The percentage of starting weight of each mouse over time ± SD. Groups are significantly different (p<0.05) on days 19–21 (mixed-effects analysis). (C) IFUs isolated from vaginal swabs over the course of infection ± SEM. Groups are not significantly different (mixed-effects analysis).

As noted above, prior studies have demonstrated that CD4 T cells and IFN-γ are each essential components of host immunity to *Chlamydia* [29,30,34,35]. It is usually assumed that this data supports a requirement for IFN-γ-producing CD4 Th1 cells in *Chlamydia* defense [11,31–33], but this hypothesis has not been directly tested. In order to examine this issue, we made use of a Cre/lox system (CD4-Cre/IFN-γ-flox/flox) [49], to explore whether IFN-γ secretion by CD4 T cells is required for *Chlamydia* clearance within the FRT. We used these mice to generate bone marrow chimeras where T cells are deficient in IFN-γ. As expected, activated CD4 T cells from wild-type mice with CD4-Cre/IFN-γ-flox/flox bone marrow had significantly reduced IFN-γ production compared to non-Cre expressing mice (Fig 8A). Despite this deficiency, CD4-Cre/IFN-γ-flox/flox chimeras resolved *Chlamydia* infection similarly to non-Cre expressing chimeras (Fig 8B and 8C) and did not develop systemic bacterial infection, as no bacteria were detected in spleen, lung, or kidney of mice on day 21 (n = 5 per group), demonstrating that IFN-γ from CD4 T cells is not an essential component of *Chlamydia* immunity in the FRT.

## Discussion

*Chlamydia* infection is often described as a Th1 disease, meaning that protective Th1 cells produce IFN-γ to restrict *Chlamydia* growth in the female reproductive tract epithelium [11,31–33]. Although this is a reasonable hypothesis, given the essential nature of CD4 T cells and IFN-γ in resolving primary infection, it has not yet been rigorously tested. Here we show that mice with CD4 T cells that lack the capacity to secrete IFN-γ still resolve *Chlamydia* infection of the FRT. This result dovetails nicely with our previous report showing that T-bet expression is not required to resolve *Chlamydia* infection in the mouse model [30]. It is further supported by experiments by Mercado et. al showing that transfer of IFN-γ-deficient CD4 T cells into TCRβ-deficient mice was sufficient to control FRT infection and protect mice from fatal infection [34]. Thus, neither Th1 lineage development nor IFN-γ production by CD4 T cells are essential components of *Chlamydia* immunity in the FRT. Together, these data make a compelling case for a reassessment of CD4-effector immunity in *Chlamydia* infection. However, it is important to note that this result differs from the mouse model of *C*. *trachomatis* infection, where CD4 T cell production of IFN-γ appears to be essential [50], likely due to differences in pathogen adaptation to different hosts between *C*. *muridarum* and *C*. *trachomatis*.

Importantly, our data still supports the idea that IFN-γ is an essential contributor to systemic bacterial clearance. Indeed, mice lacking IFN-γ or IFN-γR cannot resolve *Chlamydia* and quickly succumb to disseminated infection. However, the cells that require IFN-γ responsiveness are non-hematopoietic cells, while IFN-γR is not required on immune cells. This is somewhat unexpected since in vitro studies have shown the ability of *Chlamydia* to infect and survive within macrophages unless activated by IFN-γ [51,52]. It is formally possible that some host immune cells in the FRT are resistant to radiation and provide a niche for bacterial growth. However, the simplest interpretation of our data supports a model whereby infected epithelial cells require IFN-γ produced by innate cells as previously suggested [34,35]. Future Cre/flox studies should be able to determine which cell is required to produce IFN-γ in the FRT. It remains unclear how sensing of IFN-γ by non-hematopoietic cells would lead to containment of *Chlamydia* within the FRT, but one possibility would be that dissemination itself occurs via non-hematopoietic cells such as endothelial cells. To date, the systemic cellular niche occupied by *Chlamydia* has not been carefully delineated, especially within secondary infection sites. Recent work has shown *Chlamydia* infection of the gastrointestinal tract in wild-type mice and that IFN-γ from CD4 T cells clears *Chlamydia* from the small intestine, but not from the large intestine [53]. More work will be required to understand the role of CD4-derived IFN-γ in limiting replication within the intestine and these other disseminated sites of infection.

The exact nature of the CD4 effector module in *Chlamydia* immunity remains elusive, despite the examination of a variety of transcription factors and cytokines. From our studies, it seems clear that neither Th1 or Th2 cells play an obligate role in *Chlamydia* immunity. Thus, mice lacking T-bet, STAT6, or CD4-derived IFN-γ can fully resolve *Chlamydia* infection in the FRT. However, it is of interest that mice with a Th17 deficiency displayed a consistent delay in bacterial clearance. Given the low percentage of Th17 cells in the FRT of wild-type mice, this result was somewhat unexpected, but perhaps points to a key role for Th17 cells in coordinating neutrophil influx to phagocytose *Chlamydia* elementary bodies after rupture of infected epithelial cells. An alternative possibility is that IL-22 secretion serves as an important mediator of epithelial activation, and experiments are currently underway to test this possibility. The delay in shedding observed in RORγt mutant mice was not replicated when wild-type mice were administered neutralizing anti-IL-6 and anti-TGF-β to prevent Th17 development, perhaps due to incomplete blockade of the Th17 program. In comparing mRNA expression between CD4 T cells from infected wild-type and RORγt mutant mice, two genes with significant adjusted p-values were *Il1r1* and *Il17re*. *Il1r1* has been shown to be regulated directly by RORγt [54], confirming that the mutation in this model directly affects this regulatory pathway. *Il17re* comprises part of the receptor for IL-17C, which is produced by epithelial cells and can also be stimulated by IL-1 [55]. Similar to a mouse model of *C*. *rodentium* infection [56], IL-17C might act as an early signal for *Chlamydia* infected FRT epithelial cells. Interestingly, another differentially expressed gene was *Ramp1*, which encodes a Receptor activity-modifying protein known to modulate the signaling and trafficking of a variety of G-protein coupled receptors, including chemokine receptors [57,58]. RAMP1 can be expressed in T cells and RAMP1 deficiency alters T cell trafficking patterns [59]. Additionally, RAMP1-deficiency is linked to suppression of Th17 function and IL-17 production [60]. Thus, lower RAMP-1 expression in RORγt mutant mice might hinder responding *Chlamydia*-specific CD4 T cells from localizing to the FRT or may simply be related to lower Th17 type effector functions that assist bacterial clearance.

One intriguing outcome from this study is the severe deficiency noted in IL-12p40-deficient mice resolving *Chlamydia* infection. This is interesting because of the apparent disconnect with a previous report showing that IL-12p40 was not an essential component of *Chlamydia* immunity [44]. Indeed, we confirmed that this major deficiency in IL-12p40-deficient mice is dependent upon the strain of *Chlamydia* used for experimental studies. This opens up the possibility that other aspects of *Chlamydia* immunity are also underappreciated due to infection of mice with low pathogenicity strains. This result is also interesting because it uncovers a major deficiency due to the absence of a cytokine that typically drives Th1 or Th17 responses, yet individual or combined deficiency in Th1/Th17 subsets does not produce the same outcome. We conclude that IL-12p40 plays a critical role in *Chlamydia* immunity, but that current understanding of CD4 subset polarization does not fully explain CD4-mediated *Chlamydia* clearance. Any model of IL-12p40-dependent but Th1/Th17-independent clearance of Chlamydia is speculative at this point. However, it is of some interest that transcriptional networks downstream of IL-12R are not always linear and that T-bet-independent STAT4 activation can occur. Indeed, transcriptional activation of some genes in Th1-like cells is STAT4-dependent but independent of T-bet expression [61]. Studies are underway to assess whether STAT4 expression in CD4 T cells is required for *Chlamydia* immunity, independent of T-bet and whether some of these STAT4-dependent genes regulate additional effector pathways that are independent of IFN-γ production.

In conclusion, our data suggests that the commonly accepted Th1 paradigm of IFN-γ-producing CD4 T cells being required for *Chlamydia* clearance in the FRT is not supported by experimental evidence. Instead, we propose that *Chlamydia*-specific CD4 T cells develop an IL-12p40 dependent effector module distinct from currently accepted Th1, Th2, or Th17 subsets, but one that leads to robust defense of the mucosal epithelium. While IFN-γ remains an important contributor to this CD4-mediated defense, it is sourced from innate immune cells, sensed by non-immune cells within and outside the FRT, and largely functions to prevent systemic infection. It seems possible that IFN-γ induces epithelial expression of MHC class-II, thus allowing active surveillance of the epithelial layer by cytotoxic CD4 T cells or other effector modules. Future vaccine efforts for *Chlamydia* infections should refrain from focusing on inducing *Chlamydia*-specific Th1 or Th17 cells, since the appropriate module that allows elimination of *Chlamydia* from epithelial cells is a distinct CD4 T cell maturation pathway and remains to be identified.

## Materials and methods

### Ethics statement

This study was carried out in strict accordance with the recommendations in the Guide for the Care and Use of Laboratory Animals of the National Institutes of Health. The University of California Davis is accredited by the Association for Assessment and Accreditation of Laboratory Animal Care (AAALAC). All animal experiments were approved by University of California Davis Institutional Animal Care and Use Committee (IACUC) (Protocol number 21869).

### Mice

C57BL/6 (JAX stock no. 000664), STAT6-deficient (JAX stock no. 005977), *Tbx21*-deficient (JAX stock no. 004648), IL-12p40-deficient (JAX stock no. 002693), RORɣt mutant (JAX stock no. 031393) [62], IFN-γ R1-deficient (JAX stock no. 003288), CD45.1 (JAX stock no. 002014), IFN-γ-deficient (JAX stock no. 002287), MHC class II-deficient (JAX stock no. 003584), and CD4-Cre (JAX stock no. 017336) mice were purchased at 6–8 weeks old from The Jackson Laboratory (Bar Harbor, ME) and used for experiments at 7–12 weeks old. IFN-γ floxed mice were acquired from Dr. Harty from the University of Iowa. For many of these strains, breeding colonies were established to supply experiments. Mice were handled and used according to regulations of the Institutional Animal Care and Use Committee at University of California, Davis.

### *Chlamydia* infections

One week prior to infection, mice were given 2.5mg Depo-Provera (medroxyprogesterone acetate, Pfizer) s.c. in a 0.1mL volume. Mice received 1x10^5^ IFU of *Chlamydia muridarum* intravaginally in 5μL SPG buffer. The strain used for all experiments was derived from ATCC stock unless otherwise noted as Nigg.

### *Salmonella* infections

Mice infected with *Salmonella enteria* Typhimurium strain BMM50 (SL1344 Δ*aroA*) received 5x10^5^ CFU i.v. Stock bacterial suspensions were streaked onto MacConkey agar, from which one colony was used to inoculate an overnight culture of Luria-Bertani broth. This culture was used to prepare the infection suspension by diluting into 1X PBS for a total injection volume of 0.2mL.

### Counting *Chlamydia* burden

To monitor vaginal shedding of *Chlamydia muridarum*, vaginal swabs were taken and placed into 2mL microcentrifuge tubes containing 500μL SPG buffer and two glass beads. The tubes were shaken at 1400rpm for 5min at 4°C, and the swab subsequently discarded. Samples were frozen at -80°C until used for the counting protocol. To determine IFU burden in the organs spleen, lung, and kidney harvested from infected mice, organs were homogenized in 2mL SPG. 1mL was transferred to a 2mL microcentrifuge tube with two glass beads and samples were shaken at 1400rpm and 4°C for 5min. To remove debris from suspension, samples were centrifuged at 500g and 4°C for 10min and the supernatant removed and frozen at -80°C. To count the bacteria, samples were diluted in a series used infect a monolayer of HeLa cells in 96-well plates. These were cultured into inclusions overnight, then fixed and stained before counting to calculate IFU per swab.

### Flow cytometry

To isolate lymphocytes from lymph nodes, frosted slides were used to break up lymph nodes and washed wish PBS containing 2% fetal bovine serum. To isolate lymphocytes from the female reproductive tract, the FRT was harvested into complete RPMI, then minced into small pieces and incubated with collagenase IV (386mg/L MP Biomedicals) for 1 hour at 37°C. The resulting suspension was filtered (70μm cell strainer, Corning) and lymphocytes isolated on a Percoll gradient (GE Healthcare). For intracellular staining, cells were cultured in stimulating conditions with PMA (0.2 mM, Millipore Sigma) and Ionomycin (1μg/mL, Millipore Sigma) along with Brefeldin A (71.4μM Millipore) for 3.5 hours at 37°C 5% CO_2_. A viability stain was performed first using Zombie Yellow (BioLegend), then surface markers were stained, including B220-APC-eF780 (RA3-6B2, eBioscience), CD11b-APC-eF780 (M1/70, eBioscience), CD11c-APC-eF780 (N418, eBioscience), F4/80-APC-eF780 (BMB, eBioscience), CD4-PE (RM4-4, eBioscience), CD4-eF450 (RM4-5, eBioscience), CD8-PerCPCy5.5 (2.43, Tonbo), CD44-APC (IM7, eBioscience), and CD62L-PETexasRed (MEL-14, Invitrogen). Then intracellular stains IFN-γ-BV785 (XMG1.2, BioLegend), T-bet-PECy7 (4B10, eBioscience), RORγt-BV421 (Q31-378, BD Biosciences), and IL-17A-FITC (17B7, eBioscience) were used with the Foxp3 Transcription Factor Staining Kit (eBioscience). Data was acquired on an LSRFortessa (BD) and analyzed using FlowJo (Tree Star, San Carlos, CA). Contour plots are shown with 5% outliers.

### Bulk RNA-seq

The bulk RNA-seq experiment was performed as in the previous study [30]. For each group, five samples were prepared by pooling three individual mice each. Lymphocytes were isolated from the FRT, as described above. CD4 T cells were enriched using a negative MACS CD4 T cell isolation kit on LS columns (Miltenyi Biotech). These cells were stained for subsequent FACS sorting using the viability stain Zombie Yellow (BioLegend) and antibodies for surface markers APC-B220 (RA3-6B2, eBioscience), APC-F4/80 (BM8.1, Tonbo Biosciences), APC-CD11b (M1/70, Tonbo Biosciences), APC-CD11c (N418, Tonbo Biosciences, eF450-CD4 (RM4-5, eBioscience), and PerCP-Cy5.5-CD8a (53–6.7, eBioscience). Events passing through the sequential gates for single cell, live, dump negative (B220, CD11b, CD11c, F4/80), and CD4+CD8- were collected and processed to isolate RNA (Qiagen RNeasy Mini Kit). Gene expression profiling was carried out using a 3’Tag-RNA-Seq protocol. Barcoded sequencing libraries were prepared using the QuantSeq FWD kit (Lexogen, Vienna, Austria) for multiplexed sequencing according to the recommendations of the manufacturer using also the UMI Second-Strand Synthesis Module (Lexogen). The fragment size distribution of the libraries was verified via micro-capillary gel electrophoresis on a Bioanalyzer 2100 (Agilent, Santa Clara, CA). The libraries were quantified by fluorometry on a Qubit fluorometer (LifeTechnologies, Carlsbad, CA), and pooled in equimolar ratios. Up to forty-eight libraries were sequenced per lane on a HiSeq 4000 sequencer (Illumina, San Diego, CA) with single-end 100 bp reads to 4–7 million reads per sample. Analysis of the sequencing data and differential gene expression was performed by UC Davis Bioinformatics Core. Raw reads were processed with HTStream v.1.1.0 (https://s4hts.github.io/HTStream/) to perform raw sequence data QA/QC, remove adapter contamination and low-quality bases/sequences. The trimmed reads were aligned to the *Mus musculus* GRCm38 primary assembly genome with GENCODE v.M23 annotation, using the aligner STAR v. 2.7.0f [63] to generate raw counts per gene. Differential expression analyses were conducted using limma-voom [64] (edgeR version 3.20.9, limma version 3.34.9, R version 3.4.4). The model used in limma included effects for treatment, RNA extraction batch, number of cells, and age at death. *Mus musculus* Ensembl gene identifiers and annotations were used in this study [65]. Raw data can be accessed via the NCBI’s Gene Expression Omnibus [66] through GEO Series accession number GSE193909.

### Cytokine depletion *in vivo*

Mice were given 250μg each of anti-IL-6 (MP5-20F3, cat. BE0046, BioXCell) and anti-TGF-β (1D11.16.8, cat. BE0057, BioXCell), or isotype controls IgG1 anti-horseradish peroxidase (HRPN, cat. BE0088, BioXCell) and IgG1 unknown specificity (MOPC-21, cat. BE0083, BioXCell) i.p. starting on day 0 and every other day for 6 weeks.

### Generation of bone marrow chimeras

Recipient mice were irradiated at 800rad in an X-ray irradiator and placed on antibiotics by diluting 6mL of Pediatric Suspension Cherry Flavor (NDC 65862-496-47) into 250mL water bottles. Approximately 16 hours later, bone marrow was harvested from donor mice by flushing the marrow from femurs, tibias, and humeruses with 2.5% FBS in PBS. The cell suspension was run through 70μm cell strainers and red blood cells lysed by treating with ACK lysis buffer for 2min at room temperature before lysis was stopped upon addition of further 2.5% FBS in PBS. Cell suspensions were washed twice with PBS before being suspended in PBS for injection via tail vein. Recipient mice received 6x10^6^ donor bone marrow cells in 0.2mL total volume. Mice were kept on antibiotics for 6 weeks. To assess chimerism via congenic markers, blood was collected via retro-orbital bleeds from recipient mice at week 8, RBCs lysed with ACK lysis buffer as described above, and samples stained for flow cytometry. The markers used were CD8-BV785 (53–6.7, BioLegend), CD4-BV786 (RM4-5, BD Biosciences), CD11b-FITC (M1/70, Tonbo), CD11c-PECy7 (N418, Biolegend), B220-FITC (RA3-6B2, eBioscience), CD45.1-PerCPCy5.5 (A20, BioLegend), and CD45.2-PE (104, eBioscience).

### Statistics

Statistics were performed using GraphPad Prism version 9 (GraphPad Software, LLC). Either t-test, one-way ANOVA, two-way ANOVA, or mixed models were used. * p<0.05, ** p<0.01, *** p<0.01, and **** p<0.0001.

## Supporting information

S1 Fig Example flow cytometry gating strategy. Example gating of a representative wild-type FRT sample from Fig 2. Cells are gated sequentially on lymphocytes, single cells, negative for the live/dead stain, dump negative (B220, CD11b, CD11c, F4/80), CD4+ CD8-, CD44hi CD62L-, then either T-bet versus IFN-γ or RORγt versus IL-17A.
(TIF)

S2 Fig T-bet-deficient X RORγt mutant mice and IL-12p40-deficient mice show altered patterns of Th1 and Th17 markers. Lymphocytes isolated from the FRT and ILN were stimulated with PMA and ionomycin with Brefeldin A before staining for flow cytometry. Results are gated on CD4+ CD44hi cells. n = 3 for all groups except RORγt mutant ILN, where n = 2 as ILN were unable to be recovered in one mouse. A) Expression of Th1 markers T-bet and IFN-γ. B) Expression of Th17 markers RORγt and IL-17A. C and D) Summary graphs from A and B. E) Percentages and numbers of total CD44hi CD4 T cells. All graphs are displayed as mean ± SD. Data is representative of two experiments.
(TIF)

S3 Fig The severe delay in clearance in IL-12p40 deficient mice is dependent on the *Chlamydia* strain used, though MHC class II deficient mice do now show a difference in response. A) Wild-type or IL-12p40 deficient mice were infected with either *Chlamydia* derived from ATCC stock or Nigg strain. IFUs were counted from vaginal swabs over the course of infection. n = 3 for both wild-type groups, n = 3 for IL-12p40KO-ATCC, and n = 2 for IL-12p40KO-Nigg. For wild-type-ATCC versus IL12p40KO-ATCC p<0.05 on days 18–27. For wild-type-ATCC versus IL12p40KO-Nigg p<0.05 on day 3. For IL12p40KO-ATCC versus wild-type-Nigg p<0.05 on days 3, 12, and 22–27. For IL12p40KO-ATCC versus IL12p40KO-Nigg p<0.05 on days 3–6, p = 0.07 on day 30, and p = 0.06 on day 35. For wild-type-Nigg versus IL12p40KO-Nigg, p<0.05 on day 3. Wild-type-ATCC versus wild-type-Nigg is not significant (mixed-effects analysis). B) Wild-type or MHC class II-deficient mice were infected with either *Chlamydia* derived from ATCC stock or Nigg strain. IFUs were counted from vaginal swabs over the course of infection. n = 2 for wild-type ATCC, n = 4 for MHCII KO ATCC, n = 3 for wild-type Nigg, and n = 4 for MHCII KO Nigg. Wild-type-ATCC versus wild-type-Nigg, MHCII KO-ATCC versus MHCII KO-Nigg, and wild-type-ATCC versus MHCII KO-Nigg are not significantly different. For wild-type-ATCC versus MHCII KO-ATCC p<0.05 for days 22 and 34. For MHCII KO-ATCC versus wild-type-Nigg, p<0.05 on days 16, 22, and 34. For wild-type-Nigg versus MHCII KO-Nigg, p<0.05 on days 9–19 (2-way ANOVA). Graphs are displayed as mean ± SEM.
(TIF)
