# Supplementary figures and images for: Elimination of Chlamydia muridarum from the female reproductive tract is IL-12p40 dependent, but independent of Th1 and Th2 cells
Source: PLoS Pathog. 2024 Jan 2;20(1):e1011914. doi: 10.1371/journal.ppat.1011914 (PMC10786385; doi:10.1371/journal.ppat.1011914)

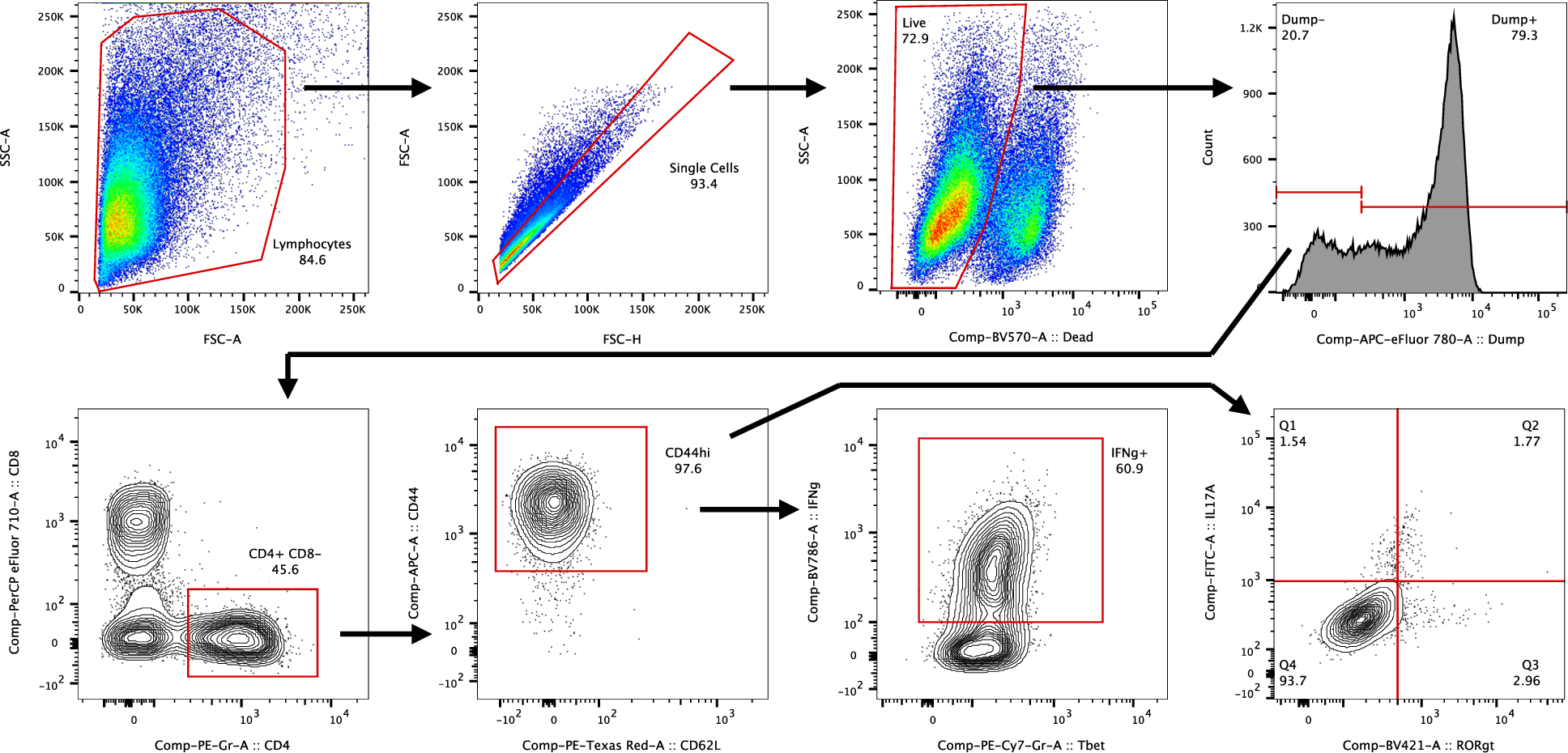

Supplement: S1 Fig — Example gating of a representative wild-type FRT sample from Fig 2. Cells are gated sequentially on lymphocytes, single cells, negative for the live/dead stain, dump negative (B220, CD11b, CD11c, F4/80), CD4+ CD8-, CD44hi CD62L-, then either T-bet versus IFN-γ or RORγt versus IL-17A. (TIF) [file ppat.1011914.s001.tif]

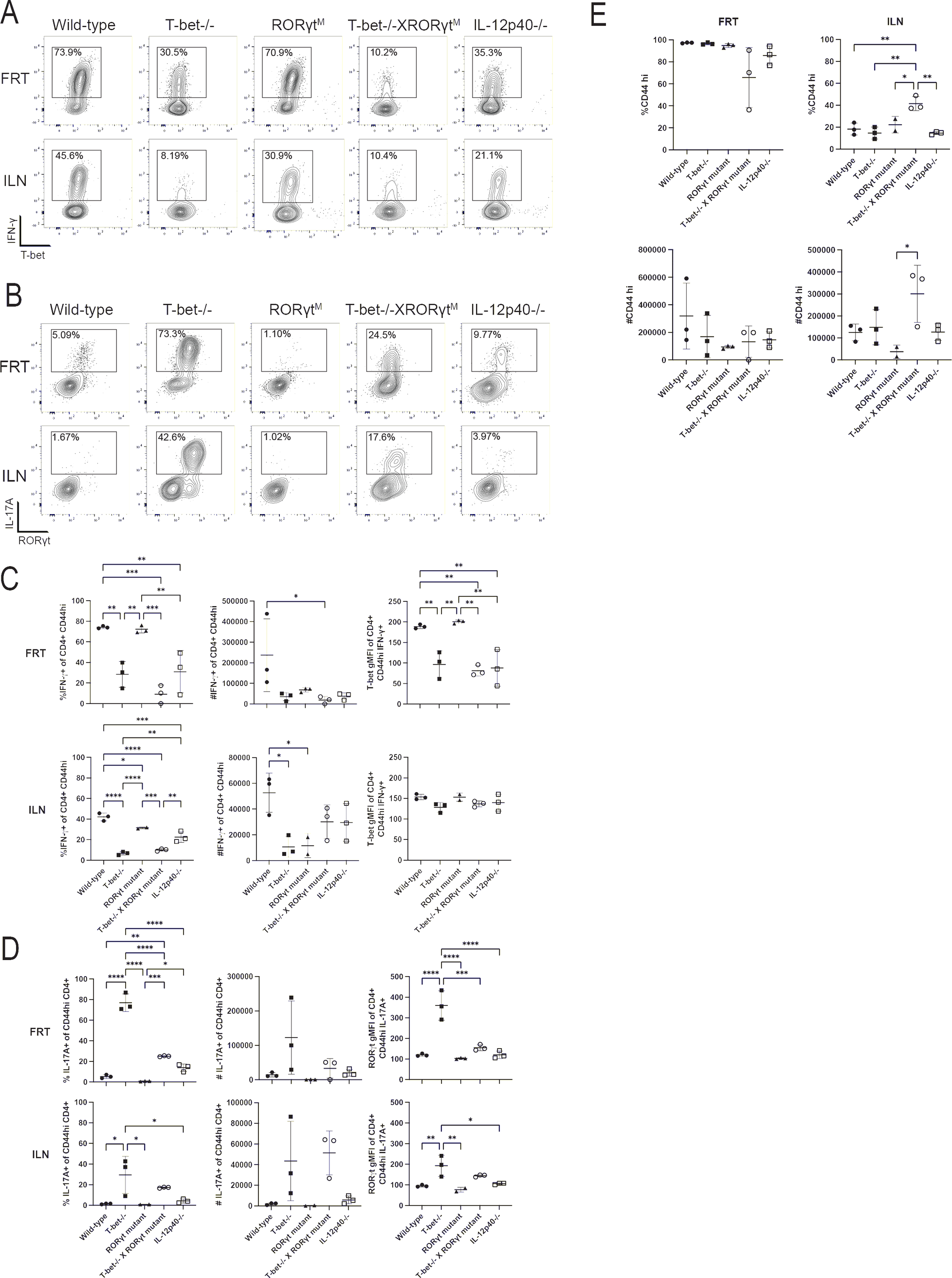

Supplement: S2 Fig — Lymphocytes isolated from the FRT and ILN were stimulated with PMA and ionomycin with Brefeldin A before staining for flow cytometry. Results are gated on CD4+ CD44hi cells. n = 3 for all groups except RORγt mutant ILN, where n = 2 as ILN were unable to be recovered in one mouse. A) Expression of Th1 markers T-bet and IFN-γ. B) Expression of Th17 markers RORγt and IL-17A. C and D) Summary graphs from A and B. E) Percentages and numbers of total CD44hi CD4 T cells. All graphs are displayed as mean ± SD. Data is representative of two experiments. (TIF) [file ppat.1011914.s002.tif]

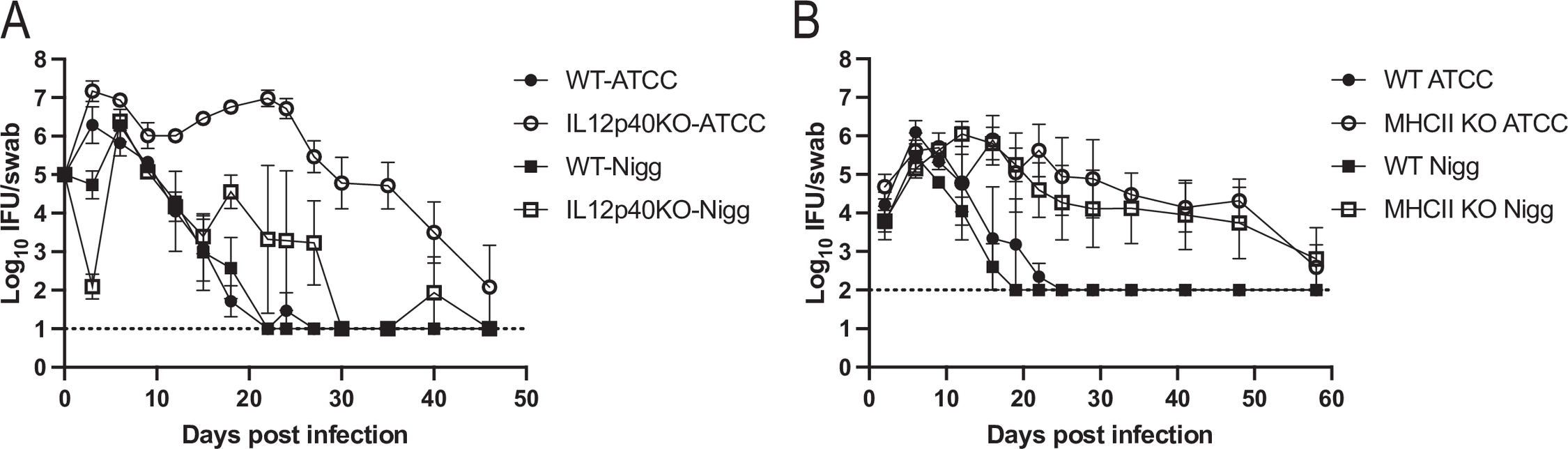

Supplement: S3 Fig — A) Wild-type or IL-12p40 deficient mice were infected with either Chlamydia derived from ATCC stock or Nigg strain. IFUs were counted from vaginal swabs over the course of infection. n = 3 for both wild-type groups, n = 3 for IL-12p40KO-ATCC, and n = 2 for IL-12p40KO-Nigg. For wild-type-ATCC versus IL12p40KO-ATCC p<0.05 on days 18–27. For wild-type-ATCC versus IL12p40KO-Nigg p<0.05 on day 3. For IL12p40KO-ATCC versus wild-type-Nigg p<0.05 on days 3, 12, and 22–27. For IL12p40KO-ATCC versus IL12p40KO-Nigg p<0.05 on days 3–6, p = 0.07 on day 30, and p = 0.06 on day 35. For wild-type-Nigg versus IL12p40KO-Nigg, p<0.05 on day 3. Wild-type-ATCC versus wild-type-Nigg is not significant (mixed-effects analysis). B) Wild-type or MHC class II-deficient mice were infected with either Chlamydia derived from ATCC stock or Nigg strain. IFUs were counted from vaginal swabs over the course of infection. n = 2 for wild-type ATCC, n = 4 for MHCII KO ATCC, n = 3 for wild-type Nigg, and n = 4 for MHCII KO Nigg. Wild-type-ATCC versus wild-type-Nigg, MHCII KO-ATCC versus MHCII KO-Nigg, and wild-type-ATCC versus MHCII KO-Nigg are not significantly different. For wild-type-ATCC versus MHCII KO-ATCC p<0.05 for days 22 and 34. For MHCII KO-ATCC versus wild-type-Nigg, p<0.05 on days 16, 22, and 34. For wild-type-Nigg versus MHCII KO-Nigg, p<0.05 on days 9–19 (2-way ANOVA). Graphs are displayed as mean ± SEM. (TIF) [file ppat.1011914.s003.tif]
